# Implementation of resilience training programmes in children and adolescents: value and barriers: Claudio Pacillo

**DOI:** 10.1093/eurpub/ckac129.523

**Published:** 2022-10-25

**Authors:** C Pacillo, W Malorni, U Moscato, LG Sisti

**Affiliations:** 1 Center for Global Health Research and Studies, Università Cattolica del Sacro Cuore, Rome, Italy; 2 Department of Woman, Child and Public Health, Fondazione Policlinico Universitario Agostino Gemelli IRCCS, Rome, Italy; 3 National Institute for Health, Migration and Poverty, Rome, Italy

## Abstract

**Background:**

Emotional distress increasingly represents a major burden in children and adolescents (C&A), especially in conflict zones where its prevalence is estimated to reach 70%. Resilience training programmes (RTPs) are interventions that seek to enhance resilience in individuals or groups pursuing mental distress prevention. Literature suggests RTPs be particularly effective in C&A; however, their effectiveness and value for public health are still unclear.

**Methods:**

A scoping review was performed in order to summarize evidence regarding the implementation and effectiveness of RTPs in children and adolescents. A search string has been built according to the PICO model and launched on PubMed, PsycInfo, Academia databases. Additional references were identified by a hand-search in Google Scholar. Studies included were narratively summarized according to topics that emerged.

**Results:**

18 articles were finally included in the review. Main issues were 1) RTPs seem to be more effective in adolescents rather than in children; 2) COVID-19 pandemic has raised the attention towards RTPs in C&A; 3) beyond conflict zones their implementation is increasingly recognized in supporting C&A management of daily stressors and traumas also in C&A with disabilities; 4) school is identified as the key setting for RTPs; 5) the high heterogeneity in RTPs approaches, contexts and study samples limits a conclusive effectiveness assessment.

**Conclusions:**

Our findings highlighted how RTPs are increasingly recognized as a tool to improve C&A cognitive and behavioral functioning and resilience to external stressors, getting greater interest in the COVID-19 pandemic. Despite relevant theoretical support and promising study results, RTPs still lack strong evidence supporting their embracement by policymakers and effective implementation in public health policy. In order to not miss this chance, more efforts are needed in strengthening RTPs conceptualization and cost-effectiveness studies.

**Key messages:**

• RTPs are a promising tool to enhance the resilience of children and adolescents gaining increasing interest due to the COVID-19 pandemic.

• More studies are needed to provide a strong evidence base that supports their acknowledgment by policymakers and their implementation in public health policies.

